# The SMART Framework: Integration of Citizen Science, Community-Based Participatory Research, and Systems Science for Population Health Science in the Digital Age

**DOI:** 10.2196/14056

**Published:** 2019-08-30

**Authors:** Tarun Reddy Katapally

**Affiliations:** 1 Johnson Shoyama Graduate School of Public Policy University of Regina Regina, SK Canada

**Keywords:** community-based participatory research, smartphones, mobile phones, population health, mHealth, eHealth, digital health, big data, evidence-based framework, citizen science, participatory research, participatory surveillance, systems science, ubiquitous tools

## Abstract

Citizen science enables citizens to actively contribute to all aspects of the research process, from conceptualization and data collection, to knowledge translation and evaluation. Citizen science is gradually emerging as a pertinent approach in population health research. Given that citizen science has intrinsic links with community-based research, where participatory action drives the research agenda, these two approaches could be integrated to address complex population health issues. Community-based participatory research has a strong record of application across multiple disciplines and sectors to address health inequities. Citizen science can use the structure of community-based participatory research to take local approaches of problem solving to a global scale, because citizen science emerged through individual environmental activism that is not limited by geography. This synergy has significant implications for population health research if combined with systems science, which can offer theoretical and methodological strength to citizen science and community-based participatory research. Systems science applies a holistic perspective to understand the complex mechanisms underlying causal relationships within and between systems, as it goes beyond linear relationships by utilizing big data–driven advanced computational models. However, to truly integrate citizen science, community-based participatory research, and systems science, it is time to realize the power of ubiquitous digital tools, such as smartphones, for connecting us all and providing big data. Smartphones have the potential to not only create equity by providing a voice to disenfranchised citizens but smartphone-based apps also have the reach and power to source big data to inform policies. An imminent challenge in legitimizing citizen science is minimizing bias, which can be achieved by standardizing methods and enhancing data quality—a rigorous process that requires researchers to collaborate with citizen scientists utilizing the principles of community-based participatory research action. This study advances SMART, an evidence-based framework that integrates citizen science, community-based participatory research, and systems science through ubiquitous tools by addressing core challenges such as citizen engagement, data management, and internet inequity to legitimize this integration.

## Population Health Science in the Digital Age

Global population health crises in the 21st century are extremely complex, with links to economic disasters [1-3], warfare [3,4], and climate change [3,5]. The digital age offers new opportunities and challenges for population health science to tackle these global crises. For instance, digital tools and technologies are increasingly being used to not only address urgent humanitarian crises [6] but also facilitate citizen participation, population health interventions, and knowledge transfer [7].

However, digital technologies are rarely evaluated for their impact on health outcomes [6], and perhaps more relevant to population health, citizens, communities, and researchers are seldom involved in designing digital tools [7,8]. Given that most digital tools are developed for profit, where privacy and confidentiality are major concerns [8,6], adoption of digital technology in population health science should take special consideration to remedy ethical pitfalls.

Given that addressing health inequities is one of the primary goals of population health science [9-11], there is a role for digital tools and technologies in contributing to the reduction of health disparities. Nevertheless, a digitally driven big data approach has the risk of widening existing health disparities if care is not taken to engage diverse populations to realize potential benefits [12]. Complex global health crises are exacerbated by health inequities and are difficult to address using traditional research practices, thus citizen science is emerging as a powerful approach that policy makers are increasingly employing in determining population health solutions [13,14].

An example of citizen science interventions is the ability to harness technology to inform active, healthy neighborhoods in developing countries [15]. This bottom-up approach of involving community members in research can also be an effective way to translate knowledge to a broader audience [14,16] and has inherent overlap with community-based participatory research, which is known to facilitate community involvement in informing research, policy, and practice [17].

Community-based participatory research has led the crusade of minimizing health disparities [18,19] by engaging communities equitably in all aspects of the research process [20]. However, global population health problems require holistic systems science solutions that enable the examination of complex interconnected factors influencing health outcomes [21,22]. As systems science has been identified as particularly effective for addressing health inequities [22], it is time now to reimagine the implementation of population health science in the digital age by developing a framework that integrates citizen science, community-based participatory research, and systems science (ie, three-pronged approach).

## Citizen Science

Citizen science enables citizens to actively contribute to all aspects of the research process, from conceptualization and data collection, to knowledge translation and evaluation [23,24]. Citizen science has its roots in environmental and ecological activism [25], where volunteers across the globe can collect data without being restricted by the constraints of traditional academic research [26].

The applicability of citizen science has become increasingly interdisciplinary over the past couple of decades [27], which has implications for population health science—a field of science that plays a key role in addressing broad health inequities [28]. Moreover, with increasing power of citizens to effect change [13], citizen science is earning a place in national science policies of countries such as the United States and Australia by complementing the efforts of governments and health professionals [14].

Citizen scientists can support and encourage regional data collection in a manner that will help researchers and policy makers to avoid the *one-size fits all* approach [14]. Involvement of citizens in research procedures is also an effective way to communicate health information to a broad audience [14,16]. Rowbotham et al [14] argue that citizen science offers an opportunity to transform population health science by engaging a larger proportion of the population in data collection to bring citizen scientist perspectives closer to traditional decision-making processes.

As citizen science can range from contributory (ie, data collection) and collaborative approaches (ie, analysis and interpretation of data) to cocreation of knowledge (ie, conceptualizing research and translating knowledge) [27], it has a natural overlap with community-based participatory research.

## Community-Based Participatory Research and Citizen Science

Community-based participatory research has a strong record of application across multiple disciplines and sectors to address health inequities [29-31]. A significant emphasis of community-based participatory research is on generating empirical evidence on social determinants of health, which in essence could be used to inform and influence population health policies.

However, as community-based participatory research is entrenched in human rights and social justice, it can be applied to promote local and regional policy change by bringing together community needs, scientific evidence, and political power [31]. In the path toward policy advocacy, participatory action can be the catalyst in engaging evidence with political power [31]. A key component in this participatory action is the involvement of diverse stakeholders in all aspects of the research process through shared responsibility and research ownership [29,32-37].

This foundational strength of community-based participatory research can result in symbiosis with citizen science in the realm of population health science, as there is considerable alignment between these 2 research approaches [38]. To translate citizen science into community voices that could potentially inform and influence policies [24], it is imperative that citizen scientist endeavors are structured using community-based participatory research principles, where citizens co-design studies and cocreate knowledge with researchers by contributing to all aspects of the research process [24,39].

As citizens are ultimately potential voters, citizen science can perhaps stimulate renewed impetus among decision makers and catalyze evidence-based policy formulation. The alignment with citizen science can catapult community-based participatory research action from local endeavors to global initiatives because citizen science is not restricted by geography, jurisdictions, or populations, thus enabling local solutions to global problems. Data collection on a global scale is the essence of citizen science, and in the 21st century, the potential for participatory modeling with a systems lens enables systems science via citizen science to solve population health issues [40].

## Systems Science, Community-Based Participatory Research, and Citizen Science

Systems science takes a holistic perspective in understanding complex mechanisms by focusing on causality and going beyond linear correlations to advance data analytics through computational modeling that could inform public health decisions [41-48]. El-Sayed and Galea [46] conclude that a systems science approach can upend traditional analytical tools with novel methods such as machine learning, microsimulation, and social network analysis that do justice to the complexity and dynamism of population health science.

Systems thinking also includes qualitative mapping and problem structuring of ill-defined issues that enable stakeholders to directly address health disparities [47]. A systems science approach for structuring problems aligns with community-based participatory research by taking into consideration the need for stakeholder involvement to tackle systemic health inequities [48-50].

Frerichs et al [41] identified 5 areas of synergy between systems science and community-based participatory research—paradigmatic, socioecological, capacity building, colearning, and translational. These synergies provide a rationale for integrating systems science and community-based participatory research, with the central concept revolving around qualitative problem-structuring and systems mapping — a process that prioritizes research questions for computational modeling to delineate the complex pathways that influence health disparities.

Systems science can change the paradigm of population health science when combined with community-based participatory research and citizen science. Apart from their interdisciplinarity, the 3 research approaches have tremendous potential for symbiosis if they are used together for reciprocal benefit. For instance, community-based participatory research-informed qualitative mapping could benefit from citizen perceptions at the population level [41], an aspect that is core to citizen science.

Nevertheless, the crux of citizen science is citizen-driven quantitative data collection [51], which has implications for both systems science and population health science. Large groups of citizens could provide individual, environmental, and social big data needed for examining interconnected, nonlinear relationships through agent-based modeling that could inform population health policies [24,52]. A systems perspective can broaden citizen science as an anchor for population health science, which is increasingly adopting socioecological frameworks [53-55]. The realization of research conducted using these frameworks requires complex multilevel subjective and objective data that enable linkages across geographic and jurisdictional barriers.

Although there are examples of citizen science projects combining social and ecological data [56-59], participation of citizens in population health endeavors (ie, large-scale linkage of individual-level health data with administrative and contextual data) has rarely been tested or implemented. Citizen science has predominantly been used for environmental change or ecological activism, whether it is reconstruction of native landscapes in disaster zones in Iraq after the collapse of Saddam Hussein or the determination of social, environmental, and economic associations in high-risk industries such as coal mining [60,61].

The bottom-up approach of citizen science to bring about social and environmental change has a place in population health science if integrated with community-based participatory research [51]. Nevertheless, the need for big data will eventually thrust systems science to the forefront in conducting population health science [62], and this need can be addressed by leveraging citizen-owned ubiquitous digital tools for large-scale data collection by combining citizen science and community-based participatory research.

## Integration Through Ubiquitous Tools

The growth of citizen science in recent years can be attributed to the global spread of internet-connected devices [63], and among all internet-connected devices, the ubiquitous presence of smartphones is the most significant development for citizen science-enabled population health research. The phenomenal growth of smartphone ownership in both developed and developing countries [64] creates an opportunity for the proliferation of citizen science across international borders.

Methodologically, the technology that powers these devices offers extraordinary research opportunities for population health science to overcome traditional constraints in terms of participant recruitment and retention, data collection and analysis, interventions, and knowledge translation [24]. Because smartphones have become a key part of day-to-day life and the primary one-stop communication device at home, work, and on the go [24,65], researchers can collaborate with citizen scientists to triangulate critical data: qualitative perceptions via audio, video, and photo-enabled ecological momentary assessments; traditional and novel quantitative data via deployment of surveys in real time; and objective data sensed via in-built smartphone sensors (eg, accelerometers, pedometers, and global positioning system) [24]. This triangulation allows consistent and longitudinal capture of big data in terms of volume, velocity, variety, and veracity [66], which is essential for the development of novel computational models informed by systems science approaches.

Although such comprehensive endeavors are not commonplace at the moment, initiatives such as the SMART Platform are leading the integration of citizen science, community-based participatory research, and systems science in implementing studies for population health surveillance, integrated knowledge translation, and policy interventions [24,67]. The success of this three-pronged approach will ultimately depend on academic leadership in engaging citizens and communities to leverage data required to address population health issues that are of concern to citizens, communities, policy makers, and researchers.

According to some estimates, there will be 6 billion smartphones in circulation by the end of 2020 [64]. Our lives may be difficult to imagine without these gadgets, and out of all the internet-connected devices that we use, smartphones are the most versatile and pervasive tools. As there is no indication that we will revert to the days without these devices, the real question is, how can we transform smartphones into effective research tools?

The three-pronged approach provides a paradigm-changing purpose to these research tools. However, for this approach to come to fruition, academia needs to play a role in empowering citizens through smartphones, especially because leaders in the technology industry do not have a strong record of safeguarding citizen privacy and confidentiality [68].

## The SMART Framework

SMART is an evidence-based framework designed to conduct population surveillance, knowledge translation, and interventions by integrating citizen science, community-based participatory research, and systems science. The framework informs the SMART Platform, which researchers are utilizing to engage with citizen scientists via their smartphones to implement multiple studies with varied study designs (eg, cross-sectional and longitudinal studies to quasi-experimental and community trials) across different jurisdictions within and outside of Canada [69]. SMART Platform provides the flexibility to engage with participants (ie, citizen scientists) in real time to capture rich population health data across jurisdictions.

Studies conducted by the SMART Platform apply mixed-methods approaches to understand not just the incidence and prevalence of health behaviors and outcomes but also where, when, how, and, more importantly *why* health behaviors and outcomes change. Comprehensive population health data collection is achieved by triangulating traditional surveys with ecological momentary assessments deployed via smartphones and mobile sensors. These data can be linked with upstream policy and administrative data and downstream health care utilization data to inform policies across jurisdictions in collaboration with primary stakeholders [24].

**Figure 1:**
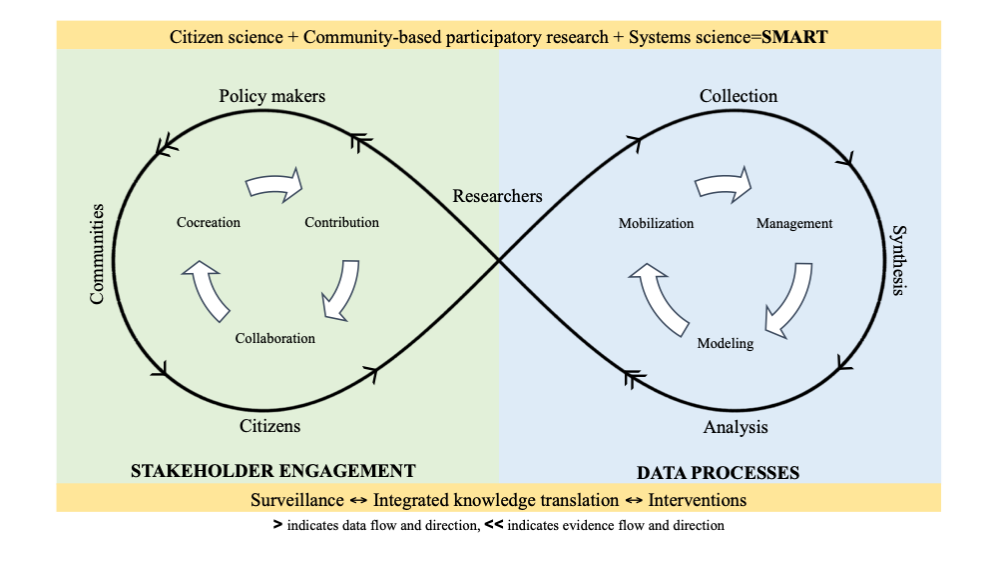
The SMART Framework: integration of citizen science, community-based participatory research, and systems science via ubiquitous tools.

The SMART Framework is rooted in continuous and consistent engagement of citizen scientists by empowering them to cocreate knowledge. The three-pronged approach allows collaborative development of research questions through qualitative systems mapping that enables data collection across the life course to inform dynamic modeling [41]. The evidence generated by this approach is translated back to all relevant stakeholders, including citizen scientists through the same devices that provide the digital data used in all analyses—smartphones (Figure 1).

Figure 1 enumerates the key components of the SMART Framework (ie, stakeholder engagement on the left side and data processes on the right side) as well as the direction of the data (>) and evidence (<<) flow in relationship with the key stakeholders. The engagement of key stakeholders in the SMART Framework is encapsulated by the interaction between citizens, communities, researchers, and policy makers [51].

The contribution, collaboration, and cocreation cycle is central to citizen science and community-based participatory research, and the engagement of stakeholders that materializes within this cycle is essential for qualitative systems mapping to determine research questions.

Researchers have a vital role to play in both empowering citizens to participate in collaboration with communities and to translate evidence back to all the stakeholders, including policy makers.

Thus, researchers occupy a central role that straddles both stakeholder engagement and data processes. The evidence translated will enable evaluation of existing research goals in collaboration with stakeholders to inform future data generation.

Data processes are driven by collection, synthesis, and analysis, with researchers playing the primary role in enabling these processes. Nonetheless, there is a significant potential for citizen involvement in data processes, depending on the level of engagement—contribution, collaboration, or cocreation [51]. These processes are dependent on data management, dynamic modeling, and evidence mobilization to ultimately convert data into evidence and translate it back to all stakeholders through the researchers.

The infinity symbol is representative of the continuous interplay between stakeholders and constant flow of data and evidence within the framework. Digital data leveraged through ubiquitous devices such as smartphones are central to this endless data generation, which is apparent from our dependence on these devices. The stakeholder interplay and data and evidence flow are facilitated by the three-pronged approach, which in turn expedites population health surveillance, integrated knowledge translation, and interventions—processes that are not only interconnected but also interdependent [24]. Overall, the framework enables quick replication of studies in different geographic locations, with an option to centralize or decentralize data collection and storage to follow ethical guidelines for data privacy, anonymity, and security.

An example of this synergy is evident from one of the initiatives informed by the SMART Framework—SMART Indigenous Youth. This initiative is a longitudinal community trial that engages indigenous youth (aged 13-18 years) and educators in rural and remote areas of Canada as citizen scientists [70]. The objective of SMART Indigenous Youth is to improve mental health outcomes among youth by embedding a land-based, culturally appropriate active living intervention into the school curricula.

As part of this initiative, youth and educator citizen scientists are able to cocreate knowledge through a strong partnership between researchers, schools, and rural and remote communities where these citizen scientists reside. The communities are independent jurisdictional units governed by Chief and Council, who also engage with researchers in concert with schools and citizen scientists. This stakeholder engagement accelerates the *big data* collection (surveillance) needed for the dynamic modeling that generates evidence translated to stakeholders (integrated knowledge translation) to evaluate the community trial (intervention).

SMART Indigenous Youth is an ideal example of research informed by the SMART Framework, and this investigation would not be possible without the power of citizen-owned smartphones. These ubiquitous tools are the reason for real-time engagement of citizen scientists in rural and remote areas, thus they serve as tools of equity for a population that is among one of the most vulnerable in Canada [71,72].

## Addressing Challenges

### Citizen Engagement: Recruitment, Retention, and Compliance

The success of citizen science is dependent on its ability to contribute data, or collaborate and cocreate knowledge with researchers [51]. However, for these actions to transpire, citizen scientists need to be engaged and empowered. Engagement and empowerment are directly connected to recruitment, retention, and compliance, which are key challenges for the success of citizen science. Through the SMART Platform [69], we are integrating citizen science and community-based participatory research to aid citizen engagement and empowerment. For example, when we recruit youth citizen scientists, we not only engage with schools and school administrators as community partners, but we also form youth citizen scientist councils, where youth interact with researchers and school administrators to drive the research process.

Currently, the SMART Platform is using mobile technology and wearables within and outside of Canada to run simultaneous surveillance and intervention studies with varied designs (eg, cross-sectional and longitudinal studies to quasi-experimental and community trials) among populations across the life course [69,70,73-76]. An important factor in maximizing recruitment, retention, and compliance is to develop strategies that are specific to different studies, cohorts, and demographic groups.

For instance, effective recruitment and retention of older cohorts (aged >65 years) requires continuous engagement and in-person deployment of mobile apps to minimize social isolation and potential technology anxiety. With respect to marginalized populations such as indigenous youth, apart from in-person group deployment through peer-to-peer interaction, incentives such as free data plans play a key role in the success of mobile health interventions.

Finally, to improve engagement from a mobile technology and methodological perspective, it is crucial to understand how mobile interfaces can be made more intuitive to reduce the burden of traditional surveys instruments. Deploying and testing ecological momentary assessments and developing replicable methods for objective data derivation are strategies used by the SMART Platform to reduce burden and increase overall compliance [69,77].

Moreover, the Platform is devised to trigger questions based on time, location, and movement to understand citizen science compliance. The challenges of citizen engagement need to be addressed by a combination of logistical, technological, and methodological solutions—before, during, and after data collection—that are facilitated by the three-pronged approach of the SMART Framework.

### Legitimization: Resources, Power Balance, and Traditional Conservatism

The SMART Framework’s three-pronged approach inherently faces challenges with legitimization, with leveraging resources being the primary obstacle, as this approach requires significant support in terms of personnel training, funding, and time [41]. Furthermore, as computational modeling processes used by researchers lack transparency, the balance of power between researchers, and communities or citizens could be another valid challenge [78].

In addition, traditional conservatism of academic research could be a larger barrier for this three-pronged approach within population health science, where there is risk of reinforcement and perpetuation of the status quo [79]. With transformative change necessitating more than the involvement of multiple stakeholders [80,81], a need exists for continuous evaluation of the three-pronged approach.

Rowbotham et al [14] state that although citizen science has been neglected by population health science, it has the potential to traverse cross-jurisdictional boundaries to provide new insights in solving population health problems. Moreover, in their study exploring the role of citizen science in transforming population health science, the authors also emphasize the role of community-based participatory research in addressing population health issues [14].

Conducting research using the three-pronged approach requires a long-term vision that cannot be restricted to short-term projects. Existing evidence indicates that citizen science projects, especially those that take a systems perspective, require longevity, and although citizen science projects have been able to obtain private and public funding for longitudinal studies through cross-disciplinary funding initiatives, there is an inherent risk of altering the project goals to meet funding requirements [82].

Utilizing the three-pronged SMART Framework, the SMART Platform has evolved into a digital methodological toolkit that can address broad population health issues ranging from the physical inactivity pandemic and youth mental health, to indigenous health and school health policies [69,70,74-76]. In doing so, the Platform has been able to secure funding from multiple private and public sources by aligning its goals with priorities of both funding agencies, and citizens and communities.

This tactic has triggered the interest of interdisciplinary researchers in citizen science–enabled population health studies, which is important for the success of the integration of citizen science, community-based participatory research, and systems science [52]. The integration also allows the transfer of power to citizens and communities, who play an important role in evaluating the implementation of the SMART Platform. In the SMART Framework, researchers empower citizens to play a larger role and challenge traditional conservatism in academia. This ultimately allows population health science to use citizen science as a tool when integrated with community-based participatory research and systems science.

### Data Management: Privacy, Security, and Linkages

Ethical considerations in terms of data privacy, security, and anonymity are at the forefront of the SMART Framework. Smartphone-enabled citizen scientist personal data provide sophisticated granularity in terms of potential identification of participants in real time through a slew of sensors such a global positioning system [83].

Protecting privacy and anonymity of citizens requires advanced encryption processes, which are embedded into the SMART Platform [24]. However, before beginning data collection and storing encrypted data, obtaining informed consent is mandatory [77]. As part of the SMART Platform, citizens not only provide informed consent through their smartphones, but they are also able to drop out of studies and request the deletion of their data. Citizens can also review the informed consent through their smartphones at any point during the study.

Beyond the ability to provide and delete data, citizen scientists in the SMART Platform co-own the data and are able to participate in data visualization, contribute to analysis, and translate knowledge—all aspects that are enabled by the integration of citizen science, community-based participatory research, and systems science. However, citizen science–enabled data need to be linked with administrative, policy, and even health care access and utilization data to address population health issues [77].

Data linkages require digital and logistical infrastructure as well as policy maker support. For citizen science engagement, technological tools that facilitate secure data collection, synthesis, analyses, and dissemination are essential [52]. These data processes require front-end human-computer interfaces such as smartphone apps and back-end interfaces that encapsulate data management, modeling, and mobilization, as articulated within the SMART Framework.

The SMART Platform utilizes sophisticated front-end interfaces, which comprise constantly evolving smartphone apps specific to various projects. This evolution is informed by the contribution of citizen scientists, communities, and policy makers. Although there is a growing movement for open-source back-end data management [84,85], the SMART Platform functions on a closed back-end database. Nevertheless, the data that are processed as part of this closed back-end are made open to public and interdisciplinary researchers after applying rigorous protocols of data anonymization. These efforts are a part of the strategy to increase interoperability through data linkages with other data sources that maximize the potential benefits of citizen science–enabled research [56,57,86-91].

The ultimate success of such data linkages is dependent on policy maker support, an integral part of SMART Framework to translate data into evidence. The SMART Platform aims to implement this approach by linking upstream behavioral citizen science data with downstream health care utilization data in collaboration with policy makers at multiple levels (local, provincial, and federal).

### Internet Inequity: Sociodemographics, Global Gaps, and Policy Initiatives

Of all the challenges that the integration of citizen science, community-based participatory research, and systems science has to overcome, the most systemic barrier is internet inequity. Internet inequity could be defined as differential access to the internet based on wealth of a country (high-, low- or middle-income), geographic region (urban, rural, or remote), socioeconomic status, gender, age, or ethnicity [12,92-94].

If the goal of the three-pronged approach is to address global population health inequities through citizen-owned devices, this integration needs to take into account the fact that there is potential for widening existing health disparities through digital divides that exclude vulnerable groups [12].

Large-scale surveys have shown significant differences in access to the internet across socioeconomic status [92,93]. Buchi et al [92] conducted structural modeling using data from representative surveys from 5 countries (New Zealand, Sweden, Switzerland, United Kingdom, and United States) to show that sociodemographics independently account for 50% of variance in usage, with age as the strongest predictor.

The Pew Research Centre’s Internet Project conducted a study to explore how smartphone dependence (ie, when one’s only means of accessing the internet is via a smartphone) and smartphone use differ between key demographic groups in the United States. Results showed that minority groups and younger, lower income, and less educated users are more likely to be dependent on smartphones. These findings suggest that smartphones can symbolize both equity and inequity depending on access to the internet [93].

The digital divide is a complex phenomenon as depicted in the study by Hilbert [94], who investigated nationally installed bandwidth potential of 172 countries from 1984 to 2014. The results indicated that internet bandwidth divide between high- and low-income countries first increased during the period of study and then decreased to historic lows between 2012 and 2014. Although there is no clear pattern in terms of the bandwidth divide across high- and low-income countries, in general, there are apparent links between bandwidth divide and income divide [94].

Although more individuals have access to global bandwidth than ever before [94], some countries are more digitally progressive and can provide the road map for better access to the internet [95]. With the United Nations declaring that access to the internet is a human right [96] and with a significant proportion (>75%) of people in 26 countries agreeing with this assessment [97], policy makers have a moral responsibility to address internet inequity.

Countries such as Canada are tackling internet inequity by allocating resources and setting national targets such as providing high speed internet to 100% of homes and businesses by 2030. This strategy is especially important in reducing the urban-rural divide in access to the internet [98].

Although it is beyond the purview of academics to address structural and systemic issues related to internet inequity, as part of the SMART Platform, researchers are engaging with policy makers, communities, and citizens to develop bottom-up approaches for providing internet access to all participating citizen scientists. For example, to provide youth citizen scientists in rural and remote areas with internet access, free data packages are being negotiated as incentives, and schools are increasing internet access to youth during and after school hours.

## Conclusions

Citizen science, community-based participatory research, and systems science need to be integrated in addressing global population health problems. However, for this integration to materialize, it is necessary to repurpose citizen-owned ubiquitous communication devices (ie, smartphones) that have revolutionized the ability to sense, share, and link big data. Smartphones have the reach to not only create equity by empowering disenfranchised citizens, but smartphone-based apps also have the capacity to source big data to inform policies through the voice of the citizens. SMART is an evidenced-based framework that integrates citizen science, community-based participatory research, and systems science through ubiquitous tools by addressing challenges such as citizen engagement, data management, and internet inequity to legitimize this integration.
